# Task-oriented training with computer gaming in people with rheumatoid arthritisor osteoarthritis of the hand: study protocol of a randomized controlled pilot trial

**DOI:** 10.1186/1745-6215-14-69

**Published:** 2013-03-09

**Authors:** Cynthia Swarnalatha Srikesavan, Barbara Shay, David B Robinson, Tony Szturm

**Affiliations:** 1 Department of Physical Therapy, School of Medical Rehabilitation, Faculty of Medicine, University of Manitoba, R106 – 771 Mc Dermot Avenue, Winnipeg, Manitoba, R3E 0T6, Canada; 2 Arthritis Centre, Health Sciences Centre Winnipeg, RR149-800 Sherbrook Street, Winnipeg, Manitoba, R3A 1M4, Canada

**Keywords:** Arthritis, Hand function, Objects of daily life, Task-oriented training, Computer games

## Abstract

**Background:**

Significant restriction in the ability to participate in home, work and community life results from pain, fatigue, joint damage, stiffness and reduced joint range of motion and muscle strength in people with rheumatoid arthritis or osteoarthritis of the hand. With modest evidence on the therapeutic effectiveness of conventional hand exercises, a task-oriented training program via real life object manipulations has been developed for people with arthritis. An innovative, computer-based gaming platform that allows a broad range of common objects to be seamlessly transformed into therapeutic input devices through instrumentation with a motion-sense mouse has also been designed. Personalized objects are selected to target specific training goals such as graded finger mobility, strength, endurance or fine/gross dexterous functions. The movements and object manipulation tasks that replicate common situations in everyday living will then be used to control and play any computer game, making practice challenging and engaging.

**Methods/Design:**

The ongoing study is a 6-week, single-center, parallel-group, equally allocated and assessor-blinded pilot randomized controlled trial. Thirty people with rheumatoid arthritis or osteoarthritis affecting the hand will be randomized to receive either conventional hand exercises or the task-oriented training. The purpose is to determine a preliminary estimation of therapeutic effectiveness and feasibility of the task-oriented training program. Performance based and self-reported hand function, and exercise compliance are the study outcomes. Changes in outcomes (pre to post intervention) within each group will be assessed by paired Student *t* test or Wilcoxon signed-rank test and between groups (control versus experimental) post intervention using unpaired Student *t* test or Mann–Whitney *U* test.

**Discussion:**

The study findings will inform decisions on the feasibility, safety and completion rate and will also provide preliminary data on the treatment effects of the task-oriented training compared with conventional hand exercises in people with rheumatoid arthritis or osteoarthritis of the hand.

**Trial registration:**

ClinicalTrials.gov: NCT01635582

## Background

Rheumatoid arthritis (RA) is a chronic symmetric polyarthritis that can affect most synovial joints, but particularly the small joints of the hands, wrists and feet. In 80 to 90% of people affected with RA, the metacarpal phalangeal and proximal interphalangeal joints of the hand and wrist joints are involved [1,2]. Osteoarthritis (OA) affecting the hand is characterized by progressive cartilage loss and associated damage to joint margins and periarticular structures in the basal thumb, proximal interphalangeal and distal interphalangeal finger joints [3]. Pain, stiffness, fatigue, joint deformity, reduced joint range of motion and hand strength in RA and OA leads to limitations in common daily activities [1,4] and profoundly restricts the individual’s ability to participate in home, work, family and community life.

In spite of the advancements in pain or disease-modifying medications and joint surgeries, many people affected with RA do experience varying levels of difficulties in activities of daily living with reduced function and quality of life [5-7]. Nonpharmacological management is recommended in both RA and hand OA as an adjunct that often includes splinting, assistive devices, exercises, self-management techniques, joint protection and therapeutic modalities. The role and therapeutic effects of exercises in RA and OA populations have been discussed in many reviews [8-15]. A 2004 systematic review that included nine studies which evaluated any form of hand exercises on pain, stiffness, range of motion, grip and pinch strength, dexterity and function reported that there is no strong evidence for or against hand exercises in people with RA [16]. Another review concluded that an optimal hand exercise program in RA is yet to be established [17].

With respect to OA, the European League against Rheumatism added that recommendations for finger range of motion exercises, grip and pinch strength exercises or thumb muscle strengthening in hand OA was limited to level 4 expert opinion [13]. A 2010 review on randomized controlled trials and cohort studies from 1986 to 2009 reported moderate evidence for exercises in improving grip strength, range of motion, hand function and pain relief [14]. Another review with only three exercise treatment studies concluded that exercises do improve grip strength and hand function but have no effect on pain and stiffness [15]. Thus, with the limited number of studies with variability in the exercises prescribed, combination of additional therapeutic modalities, different outcome measures, exercise parameters (frequency, intensity, duration) and other methodological issues, it is difficult to arrive at definitive conclusions on the effects of range of motion or strength exercises on hand function in both populations. Although no strong evidence is available for the effectiveness of hand exercises, the theoretical basis and the purpose of prescribing exercises is to maximize function by preventing joint deformities, improving joint movement and strength of the grip muscles.

Most of the hand exercise programs prescribed for people with arthritis commonly include a variety of exercises aimed to improve the range of motion of the hand and wrist joints and/or to improve/maintain grip and hand intrinsic strength. However, other components of hand function such as dexterity are often not considered in hand rehabilitation programs [18]. In contrast, task-specific training with manipulation of common objects could also be incorporated for effective transfer into activities of daily living [18-20]. Low compliance and treatment adherence are the significant hurdles to be overcome in long-term exercise programs, especially in chronic illnesses such as arthritis. As a result, there has been an additional need for rehabilitation programs that provide motivation in order to continue and complete treatment regimes. Maximizing individual participation is seen as a main goal of any intervention that is central to functional success.

Since the 1980s, computer games have been used for therapeutic purposes in different patient populations [20-26]. Several recent studies have described the use and benefits of interactive computer games and it has been reported that they provide interesting and challenging activities [23], and are enjoyable, engaging and intrinsically motivating [20,23,25]. Utilizing biofeedback and motion-sensor technology advancements, an innovative, computer-based gaming platform interfaced by a commercial motion mouse that combines interactive computer games with handling and manipulation of day-to-day objects has been developed. A novel, task-oriented training program has also been designed that is aimed to extend the clinical rehabilitation experience to a home-based setting. The program utilizes an interface that allows use of many different common objects with diverse functional demands and physical properties to be incorporated into a personalized therapy program targeting dexterity, graded finger mobility, endurance and hand strength training. This opens up an exciting possibility for affordable and scientifically motivated improvements in arthritis rehabilitation.

Dexterity skills involving handling and manipulating objects with the fingers and hand are important for nearly all activities of daily life, such as dressing, grooming, eating, use of utensils and implements, and participation in play, hobbies and chores. These activities require manipulation of objects with a wide range of physical properties (size, shape, weight, inertia) and often require a high degree of precision, where small deviations in timing or endpoint accuracy and positioning/orientation of the object leads to complete disruption of performance. In a study by Guzelkucuk and colleagues on 36 young adults with hand injuries, a hand exercise program that included passive, active range of motion and strengthening exercises was compared with training of targeted functional activities such as using a spoon, rolling a cylinder, and locking and unlocking a door key [18]. Improved hand function was reported in the group that received these task-specific functional activities. The authors also concluded that activities aimed at dexterity should be incorporated into hand exercise rehabilitation, which would increase the transfer to life role participation. Building on a similar concept, the novel training program will adopt the following principles of task-oriented approach [19,27]: functional movements directed towards normal day-to-day activities, hobbies and work via manipulation of real-life objects; personalized treatment goals and training loads based on individual needs and abilities; rehabilitation focused on acquisition of skills and active patient participation for performance of meaningful and relevant tasks of daily life; and repetitive training to increase endurance and intensity of practice. In consensus with the general opinion and current practice of the use of mobility exercises in the management of RA/OA affecting the hip, shoulder and knee [28-30], we also expect that exercises involving graded mobility of the joints of the hand and wrist could improve hand function and reduce pain and stiffness in people with RA or OA of the hand. Exercise repetition as tolerated, endurance and strength are also other key components of the program.

**Figure 1:**
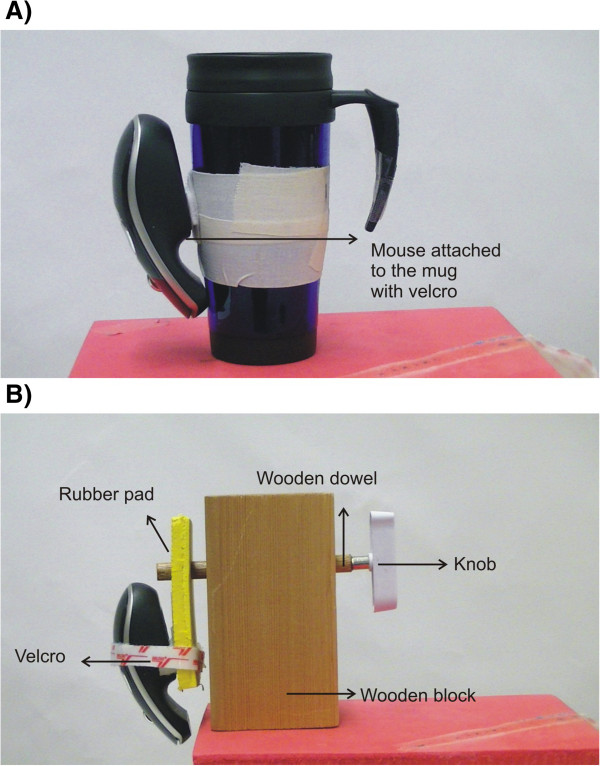
**Instrumentation of objects.** (**A**) A coffee mug is instrumented with the motion sense mouse using Velcro strips. (**B**) A turning knob is instrumented with the mouse using a wooden block, dowel, rubber pad and Velcro strips.

Modern concepts of training using a task-specific approach are achieved by incorporating a wide range of common objects/tools/utensils used in daily life activities. Specifically, the therapy consists of manipulating common daily objects that have been instrumented with a motion sensor which seamlessly transforms each object, tool or utensil into a computer input device. A low-cost motion-sense mouse has been chosen for this purpose (Gyration air mouse Elite; SMK-Link Electronics, Camarillo, CA, USA). This mouse is small with gyroscopic and accelerometer sensors that are used to derive angular displacement signals. The motion signals are used in a manner identical to a computer mouse to control onscreen cursor motion. The motion mouse is secured by Velcro to many different objects, and this simple method allows object natural motions to be used as the computer input device. This allows manipulation of each object to play nearly any computer games. A wide range of common objects can be instrumented with the mouse. Most of the objects can be directly instrumented (Figure 1A) and a few small-sized objects require a slightly modified structural set up (Figure 1B).

**Figure 2:**
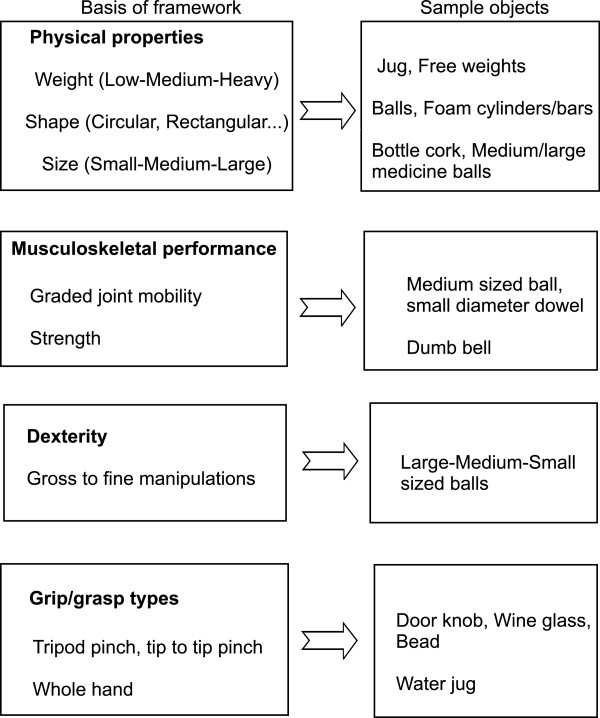
**Framework for selecting personalized therapeutic objects for the task**-**oriented training program.**

Common objects of daily life have different physical, functional and ergonomic properties. Taking advantage of these properties, objects can be selected to train dexterity, graded mobility in metacarpal phalangeal, interphalangeal and wrist joints, or strengthen finger and wrist muscles. A framework (Figure 2) has been developed for selecting therapeutic objects for specific therapeutic values. The application allows manipulation of objects of any shape (cylindrical, spherical, conical and rectangular) or size (small, medium and large). A wide range of small to large objects can be used to train dexterous functions involving the use of two, three or four fingers or the whole hand, wrist and forearm in order to improve task accuracy, precision and movement quality/efficiency. Training of such skills are expected to emulate many daily functional activities such as holding/manipulating fine, medium and large objects, and cylindrical or spherical objects, turning a door key, doing up buttons, or pulling up/down zippers during dressing. A bottle cork, salad tongs, door knob, key, and fine beads are a few objects that can be selected to train fine manipulations. Large-sized balls, large–medium-sized scissors, a coffee mug, large–medium–small diameter paper rolls and sponge foam cylinders can be selected for gross manipulations. In addition to dexterity, strength training needs to be included because hand strength is also needed to perform many daily tasks requiring a strong grip, such as carrying heavy items, opening tight jars and turning large knobs.

Any commercially available computer game that requires mouse movements in the *x*, *y* or both axes can be used in the training program. Many commercial computer games are inexpensive, suitable for all ages and are easy to use. Computer games have tremendous potential as rehabilitation tools because they provide a wide range of challenges in precision level, movement speed, amplitude and direction while playing. Added features such as distracters, reaction time, strategies and difficulty levels, visual–spatial and cognitive challenges complement the basic game mode for more fun and motivation. However, it is important to identify games that suit the preferences and match the functional abilities of each individual. Games should also be simple, easily understandable and well balanced between skill, chance and strategy. Skill should be able to bring the best of the required rehabilitative movements, while chance and strategy keep the individual motivated. Also important is a large and ever-changing variety of games in order to maintain high levels of motivation and interest in participating individuals. Having easy access to a large number of inexpensive commercial games makes this possible. There are a number of online sites that have a large and diverse collection of games – for example, Big Fish Games, games2download. More than 100 computer games have thus far been tested and found suitable for this novel training program.

To date, no study has reported the use of task-oriented approach in people with arthritis affecting the hand. A pilot randomized controlled trial has therefore been planned to determine the preliminary estimation of the therapeutic effectiveness and to evaluate the feasibility of the task-oriented training in people with RA or OA of the hand.

### Hypotheses

The first hypothesis is that the experimental group receiving task-oriented training will show greater improvements in performance-based, self-reported hand function and reduction in pain and stiffness levels as compared with the control group receiving conventional hand exercises.

The second hypothesis is that the task-oriented training will be feasible in terms of compliance and treatment safety and will demonstrate better completion rate as compared with conventional hand exercises.

## Methods

### Study design

The study is designed as a single-center, parallel-group, assessor-blinded randomized controlled pilot trial.

### Inclusion criteria

Men and women aged between 30 and 60 years diagnosed with RA according to the American College of Rheumatology 1987 classification criteria or symptomatic OA of the hand according to American College of Rheumatology criteria will be recruited. They recruits should also be willing to give written informed consent, own a home computer and have a basic working knowledge of computers. A moderate level of difficulty perceived in performing certain activities of daily life, with a Disability of the Arm, Shoulder and Hand (DASH) score range of 25 to 50 out of the maximum score of 100, will be an added criterion.

### Exclusion criteria

People will be excluded if they present with any of the following features: recent surgeries (<6 months) in the dominant hand; problems with vision or hearing; recent changes in drug regimen <3 months; major diseases of the heart, lungs or liver; fixed finger joint deformities; and DASH scores <25 or >50.

### Ethics approval

The study protocol was approved by the University of Manitoba Human Research Ethics Board (reference number: H2012:182).

### Sample size calculation

In this pilot trial, a formal sample size calculation was not carried out. Thirty participants [31] will be recruited and allocated into two groups throughout the data collection period.

### Interventions

#### Control group

Based on previous studies [3,32-37], a conventional hand exercise program targeted to improve finger range of motion and hand strength has been compiled. The exercises of the conventional hand exercise program are: making a full fist by flexing the metacarpal phalangeal, proximal interphalangeal and distal interphalangeal joints of all fingers; making a small fist by flexing only the proximal and distal interphalangeal joints of all fingers; flexing and extending the wrist; touching the tip of each finger with the tip of the thumb; spreading the fingers as much as possible and closing them; raising the fingers as much as possible; hand intrinsic strengthening with 85 g medium-resistance therapeutic putty; and hand strengthening with a dumbbell.

#### Experimental group

**Figure 3:**
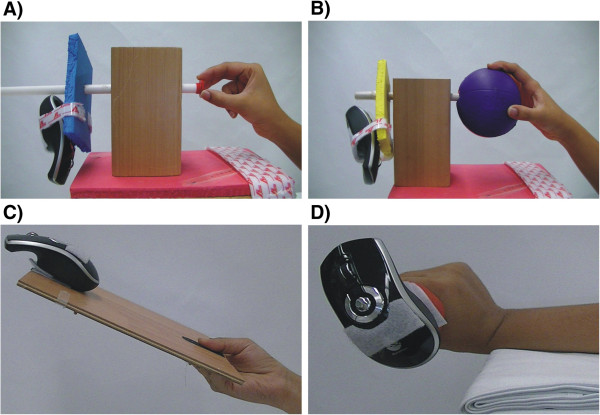
**Task**-**oriented training with a few selected objects.** (**A**) A bottle cap is manipulated by rotating it left or right. (**B**) A medium-sized ball is manipulated by rotating it left or right. (**C**) A flat wooden board is manipulated by tilting it up down and *vice versa*. (**D**) A dumbbell is used for strengthening the wrist extensors.

The task-oriented training program is targeted to improve hand function during everyday tasks requiring dexterity functions and strength. Training with a few different objects with specific therapeutic values is listed below and is illustrated in Figure 3. The random manipulative movements described for each object task will be used to control the motion of the computer game cursor.

##### Therapeutic values: precision skill training

The following involve training of fine finger manipulations using small objects and precision grips. The manipulations are within the digits involving fine mobility in the joints moved.

1. Object selected: a door key

Method: the key is held between the pad of the thumb and the lateral side of the index finger and the task requires rotating the key to the left or right.

2. Object selected: a bottle cap

Method: the bottle cap is held between the pad of the thumb and the index or middle fingers and the task requires rotating the cap to the left or right (Figure 3A).

##### Therapeutic values: manual dexterity

The following involve training of gross finger hand manipulations with medium–large objects and power grasps. The manipulations involve use of the whole hand and the palm involving graded mobility at metacarpal phalangeal, proximal interphalangeal and distal interphalangeal and wrist joints.

3. Object selected: a sport ball (size of a tennis ball)

Method: the ball is held with the whole hand and the task requires rotating the ball to the left or right direction (Figure 3B).

4. Object selected: a plate or a lightweight flat wooden board

Method: the plate/board is held with the whole hand and the task requires tilting the plate/board up or down (Figure 3C).

5. Object selected: a drinking glass

Method: the glass is held with the whole hand and the task requires rotating the glass forward down or up straight.

##### Therapeutic values: strengthening

6. Object selected: half-filled jug

Method: the jug is held with the whole hand around the handle and the task requires tilting the jug sideways up or down, simulating pouring activity.

7. Object selected: a dumbbell

Method: a dumbbell held with the whole hand and forearm supinated or pronated can be used for graded strengthening of wrist flexors or extensors (Figure 3D).

### Outcome measures

#### Primary outcome measure

The primary outcome measure will be the Arthritis Hand Function Test, an 11-item performance-based test that measures grip and pinch strength, dexterity, applied strength and dexterity in people with arthritis. Test–retest reliability of the Arthritis Hand Function Test measured by the intraclass correlation coefficient in 20 individuals with RA ranged from 0.53 to 0.96; inter-rater reliability ranged from 0.89 to 1.0. Test–retest reliability in 26 individuals with OA ranged from 0.7 to 0.96; inter-rater reliability ranged from 0.99 to 1.0 [38,39].

The peg-board dexterity test measures the time taken in seconds to place and remove nine pegs into the nine holes. Applied dexterity is the total time taken (seconds) to complete the following tasks: lacing a shoe, tying a bow, fastening/unfastening four buttons, fastening/unfastening two safety pins, cutting putty with a knife and fork, and manipulating coins into a slot.

Applied strength will be measured by the total number of soup cans lifted in a tray and the volume (ml) of water lifted in a pitcher.

Grip strength of the dominant hand will be clinically tested using a handheld dynamometer (Biometrics isometric hand dynamometer G100; Biometrics Ltd, Cwmfelingach, Gwent, UK). Grip strength will be recorded in kilograms and the best score out of the three consecutive trials will be used for analyses. Similarly, three-point pinch strength will be measured using a pinch meter (Biometrics precision pinch meter P200; Biometrics Ltd).

#### Secondary outcome measures

The secondary outcome measures are the DASH questionnaire and an exercise log diary.

The DASH questionnaire, a reliable and valid outcome measure in the arthritis population [40,41], will be used to measure self-reported hand function ability. The DASH has 30 disability/symptoms questions on difficulties in activities of daily living and scores range between 0 and 100. Higher scores indicate severe disability.

A personal exercise log diary will be used to measure exercise compliance. The number of completed exercise sessions out of 24 total exercise sessions in 6 weeks will be calculated. Any adverse symptoms such as an increase in pain or stiffness experienced due to exercising, technical difficulties reported by the participants and the study completion rate will also be documented

A computer-based paddle game with two modes – predictable visual tracking and random play – has been developed to objectively quantify the quality and accuracy of finger hand movements during manipulation of a broad range of common objects independent of physical properties, anatomical requirements and task goal/context [42,43]. For the present study, the tool will be designated as an exploratory outcome measure.

For the predictable visual tracking, predictable, sinusoidal visual tracking (configurable amplitude and frequency) provides a controlled visual input to guide a motor task. An instrumented object will be held by a specific grasp and moved rhythmically in concert with the moving visual target on the computer screen. The position coordinates of the onscreen moving target cursor and the actual object motion will be synchronously logged and saved. The data will be then processed and analyzed using custom analysis routines written in Matlab (The Math Works, Natick, MA, USA).

**Figure 4:**
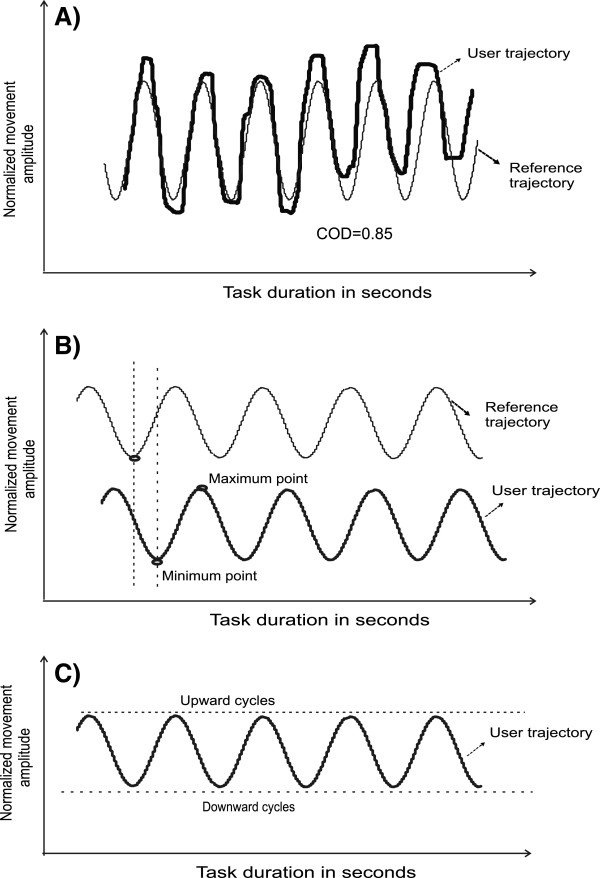
**Objective variables of the predictable tracking task.** (**A**) Coefficient of determination of user motion trajectory. Bold lined waveform, user motion signal; thin line, reference waveform for a manipulation task. (**B**) Temporal accuracy. Bold lined waveform in the top plot, reference waveform; light shading, user motion signal for a manipulation task. A maximum and minimum point on the up and down cycles of the user signal is shown. The time taken to reach maximum and minimum points for each movement cycle is compared between the two signals. (**C**) Amplitude consistency. An arbitrary line drawn on a user motion signal represents amplitude consistency in upward and downward directions.

Quality and accuracy of finger hand movements during tracking will be measured by spatio-temporal accuracy outcomes. Based on the known target trajectory and the user motion, the coefficient of determination will be calculated (Figure 4A). The coefficient of determination determines the overall movement quality of the manipulation task and the values range between 0 and 1. Values near to 1 indicate close fits of the user motion signals with the target cursor motion, and values far from 1 explain the least fit.

Temporal and amplitude accuracy measures will be determined at the turning points of each cycle of the user motion trajectory (Figure 4B,C). For temporal accuracy, the time taken to reach maximum and minimum points of each cycle of the user motion will be subtracted from the time of the respective target cursor motion maximum and minimum. Average absolute temporal accuracy (in milliseconds) of maxima and minima for all of the cycles will thus be determined. The amplitude consistency of the object motion will be determined by the coefficient of variation of the average movement amplitude in both directions.

For episodic (random) play, the goal of this game mode is to move the paddle (game sprite) to hit the random targets moving vertically top to bottom or horizontally from left to right of the computer screen. The game will be played using an instrumented object that generates a logged game file to record the time index and coordinates of each game target and to record the position coordinates of the game paddle that slaves to player’s movements (representing the respective object manipulation task). The data will then be processed and analyzed in Matlab.

**Figure 5:**
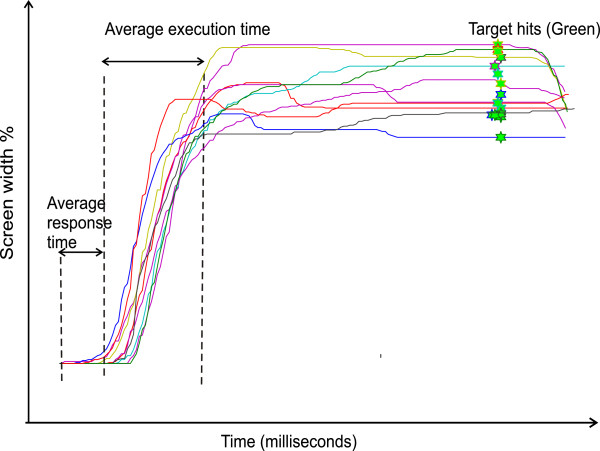
**Parsed movements from a random game play.** Outcome measures derived from episodic game play are shown.

Each one minute of game play can provide around 30 to 40 player movements (multiple events). These movements can be parsed and sorted by movement direction and movement amplitude/speed. Figure 5 shows the parsed movements in a gaming session of 2 minutes. Different features of the player’s movements provide a basis for objective quantification of variables such as: number of hits/misses of the paddle on the random targets; time measured between the appearance of the target to the start of the paddle movement (motor response time); time taken to reach the target location from the paddle initial location (movement execution time); and movement path length from start to end of all gaming events.

The tool is also embedded with two separate numerical verbal pain and stiffness scales. The pain scale ranges from 0 (no pain) to 10 (unbearable) and stiffness from 0 (none) to 10 (cannot move). These scales will appear in sequence on the computer monitor before and immediately after each pre-timed sinusoidal tracking or the random game. Participants will be asked to verbally rate their pain and stiffness intensity before and after each manipulation task. The study staff record them, and they will be automatically saved along with the user motion data.

The tool will be used to evaluate quality and spatio-temporal accuracy during four manipulation tasks with objects such as a salad tongs, a small bead, jug and a turning knob. These objects are selected to represent a diverse range of manipulation tasks needing different functional requirements of handling. Note: in order that there is no practice effect, these four objects will not be included for the experimental group. Our previous pilot work has reported that the jug and salad tongs were difficult to manipulate in people with RA or OA. So these two manipulation tasks will be evaluated in predictable tracking mode with a duration of 30 seconds. Skill and efficiency of the other two manipulations will be evaluated while playing the random game for 90 seconds. Evaluation of all four tasks may take approximately 7 to 10 minutes.

The measures derived from both game modes of the computer-based hand assessment tool are as follows: predictable tracking – task performance (coefficient of determination), spatio-temporal accuracy measures (temporal accuracy, amplitude consistency), pain and stiffness intensity; and episodic (random) game – success rate (number of target hits), movement skill (average motor initiation time, average motor response time), movement efficiency (movement path length), and pain and stiffness intensity.

### Study protocol

**Figure 6:**
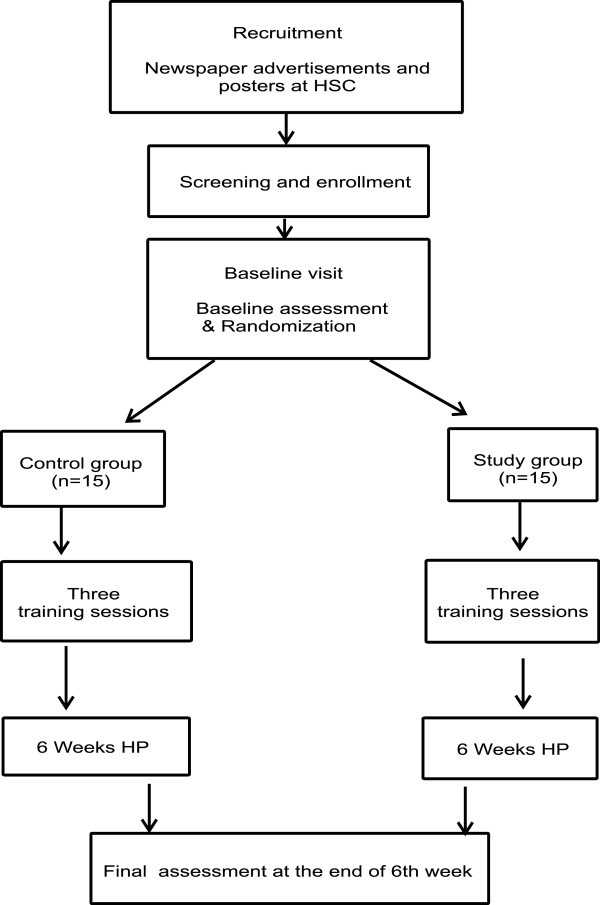
Flowchart of the clinical trial from recruitment to final assessment stages.

The sequence of events of the randomized controlled trial is illustrated in Figure 6.

### Recruitment

Thirty participants will be consecutively recruite through advertisements in local newspapers and posters at the clinic offices of the Health Sciences Center, Winnipeg. Volunteers who contact the study staff will be screened according to the study participation criteria. Eligible participants will be issued a general information handout that explains the study purpose and the need to attend a baseline session, three training sessions and a final follow-up session at the Department of Physical Therapy, University of Manitoba. Written informed consent will be obtained from each participant.

### Baseline visit

An initial assessment will be conducted during the first visit, which will include the following: documentation of demographic information such as age, sex, occupation, diagnosis and disease duration; documentation of individual specific problems during specific tasks of daily living; observation of fine/gross finger abilities on handling different objects of daily life; and assessment of primary and secondary study outcomes.

### Randomization

After the baseline assessment, participants will be randomly allocated into one of the two groups. The randomization procedure will be as follows: sequence generation, the two groups will be generated with equal allocation ratio (1:1) by referring to a computer-generated group allocation –randomization will also be stratified, RA or OA condition wise, depending upon the number of participants being recruited; allocation concealment, allocation will be concealed in sequentially numbered, opaque, sealed envelopes and will be opened only after the participant details are written on the envelope; implementation, a staff member who is not involved with the trial will generate the random numbers, do the allocation concealment and keep them until the end of the study; and blinding, an independent assessor blinded to the group allocations will evaluate the study outcomes at baseline and at the end of the sixth week.

### Home program training sessions

Based on clinical experience and pilot data, three training sessions for approximately 45 to 60 minutes each time are essential before initiating the home program. Each participant from both groups will attend the sessions within 7 to 10 days after the baseline visit.

Care will be taken to ensure that each participant is educated well to perform their respective interventions and to record pain and stiffness levels using computer software. The experimental group will also be shown how to instrument personalized objects with the mouse and how to access the computer games. All participants will be given an opportunity to discuss, clarify and ask questions regarding their home program.

The control group will be provided with medium-resistance therapeutic putty (85 g) and a dumbbell as home program accessories. The experimental group will be provided with an equipment package that includes a motion-sense mouse, a set of personalized objects and commercial computer games, and a few accessories such as Velcro strips and wooden dowel for object instrumentation. Both groups will be provided with software for use on their home computer to record and store pain and stiffness levels using separate 0 to 10 numerical verbal rating scales, before and after each exercise session.

### Six-week home program

After the training sessions, both groups will be provided with a written protocol of their home program. The protocol will include clear description of each exercise/manipulation to be performed, number of repetitions (control group) or minutes of play (experimental group) and the number of sessions per week.

The control group will perform the conventional hand exercise program, and the experimental group will perform the task-oriented training with personalized objects, selected by the clinical judgment drawn from the baseline findings.

At the start of the home program, the study staff will attend each participant’s home to ensure proper set-up of their respective intervention. A week later, both groups will be contacted through telephone calls or electronic mails to assess their ability to manage the home program. Both groups will be asked to perform their exercises four times per week for 6 weeks. The pain and stiffness files can be saved to a USB flash drive or sent as an email attachment for regular monitoring by the study staff.

Treatment progression or modification will be made for both groups every 2 weeks depending on individual needs and abilities. When no increased symptoms (assuming a benchmark score >5 in the pain and the stiffness scales) are reported during or after exercises, progression will be followed as in Table 1. In cases of any symptoms being reported, the treatment parameters will be modified by reducing the number of repetitions or minutes of play. Any occurrence of increased pain or stiffness continuing over 1 week would be recorded as an adverse event. The intervention will be discontinued and the participant will be referred to his/her general practitioner.

**Table 1:** Treatment progression guidelines for both groups

**Group**	**Progression goals**	**0 to 2 weeks**	**3 to 4 weeks**	**5 to 6 weeks**
Control group	Number of repetitions of each exercise	5	8	12
Experimental group	Personalized objects	3 to 4	4 to 5	5 to 6
Both groups	Strengthening (60 to 70% of one repetition maximum	1 set ×10 repetitions	1 set ×15 repetitions	1 set ×20 repetitions
Both groups	Exercise duration	15 minutes	20 minutes	25 minutes

The study staff providing the interventions will maintain a record of treatment sessions, exercises prescribed and their progression for each participant. Information on adverse symptoms would include severity, duration and the appropriate treatment modifications done.

A final session to evaluate the primary and secondary study outcomes will be conducted at the end of the 6th week of the home program.

### Statistical analysis plan

Normality of all data will be tested using Shapiro–Wilk’s test, based on which data will be appropriately described. Continuous variables will be reported as means ± standard deviation and noncontinuous variables will be reported as the median and interquartile range. Unpaired Student’s *t* test or Mann–Whitney U statistics will be used to compare the demographic variables and baseline outcomes between the two groups. Based on the data distribution of the study outcomes, grip strength, peg-board dexterity, applied dexterity and applied strength (Arthritis Hand Function Test items), DASH and number of exercise sessions completed in 6 weeks, changes over time (pre to post intervention) within each group will be assessed by paired Student *t* test or Wilcoxon signed-rank test. Differences in study outcomes between groups (control versus experimental) post intervention will be analyzed using unpaired Student *t* test or Mann–Whitney *U* test. An intention-to-treat analysis will be performed by including all the participants regardless of adherence to the study protocol. The last-observation-carried-forward method will be utilized to minimize the number of missing values due to dropouts. Statistical significance will be set at the *P* <0.05 level (two-tailed) for all analyses. The Consolidated Standards of Reporting Trials statement 2010 flow diagram will be used to explain the stages of the randomized controlled trial

## Discussion

The overall aim is to provide a seamless home-based hand intervention well suited to address the personalized needs and treatment goals of people with arthritis affecting the hand. The study findings will contribute to new clinical knowledge and discussion on the initial estimation of the treatment outcomes and feasibility of the novel training program in people with RA or OA of the hand. The results may also help to support the potential applicability of the home-based task-oriented training in other patient populations – for example, stroke, spinal cord injury and children with neurodevelopmental disorders affecting hand–arm function.

However, there are a few issues that may impact the interpretation of the study findings. To minimize the presence of any comorbid conditions associated with aging, the age range of the study participants included in the present study is between 30 and 60 years. Although this may be a younger age group than many clinic populations, if there is failure to find an effect in this age group it is less likely that an effect will be found in an older group. A hypothetical DASH score range of 25 to 50 out of the maximum score of 100 was selected to include individuals presenting with the perception of a moderate level of difficulty in performing common activities of daily life. We chose to exclude individuals with mild difficulties or with severe limitations because the object manipulation tasks with computer gaming (experimental group intervention) may either be very easy or difficult to perform, influencing the treatment responses. Additionally, the DASH score inclusion criterion will include people presenting with a homogeneous baseline level of self-reported symptoms and level of difficulty in activities and participation, irrespective of the type of arthritis. People with a diagnosis of heart or lung disease that may functionally debilitate the individual in normal day-to-day life are also excluded since the home-based hand exercise program may be even more demanding. As this study was designed to test the feasibility and obtain preliminary data on the effectiveness of the task-oriented training, no formal measures of disease activity were incorporated to interpret the effects of the interventions. Although this may limit the generalizability of the results to individuals with RA or OA that are relatively healthy, this is appropriate in a pilot study.

## Trial status

Ongoing.

## Abbreviations

DASH: Disability of the Arm, Shoulder and Hand; OA: osteoarthritis; RA: rheumatoid arthritis.

## Competing interests

The authors declare that they have no competing interests.

## Authors’ contributions

CSS, BS, DBR and TS have substantially contributed to the conception and design, data collection and analysis procedures; have been involved in drafting the manuscript and revising it critically for important intellectual content; and have given final approval of the version to be published.

## References

[B1] Horsten NC Ursum J Roorda LD van Schaardenburg D Dekker J Hoeksma AF Prevalence of hand symptoms, impairments and activity limitations in rheumatoid arthritis in relation to disease duration J Rehabil Med 2010 42 916 921 10.2340/16501977-0619 21031287

[B2] Philips CA Rehabilitation of the patient with rheumatoid hand involvement Phys Ther 1989 69 1091 1098 2685847 10.1093/ptj/69.12.1091

[B3] Rogers MW Wilder FV Exercise and hand osteoarthritis symptomatology: a controlled crossover trial J Hand Ther 2009 22 7 10 discussion 19–20; quiz 18 10.1016/j.jht.2008.09.002 19013758

[B4] Van der Giesen FJ Nelissen RG Rozing PM Arendzen JH de Jong Z Wolterbeek R Vliet Vlieland TP A multidisciplinary hand clinic for patients with rheumatic diseases: a pilot study J Hand Ther 2007 20 251 260 quiz 261 10.1197/j.jht.2007.04.004 17658419

[B5] Vliet Vlieland TP Non-drug care for RA – is the era of evidence-based practice approaching? Rheumatology (Oxford) 2007 46 1397 1404 10.1093/rheumatology/kem149 17586864

[B6] Vliet Vlieland TP van den Ende CH Nonpharmacological treatment of rheumatoid arthritis Curr Opin Rheumatol 2011 23 259 264 10.1097/BOR.0b013e32834540fb 21346575

[B7] Astin JA Beckner W Soeken K Hochberg MC Berman B Psychological interventions for rheumatoid arthritis: a meta-analysis of randomized controlled trials Arthritis Rheum 2002 47 291 302 10.1002/art.10416 12115160

[B8] Forestier R Andre-Vert J Guillez P Coudeyre E Lefevre-Colau MM Combe B Mayoux-Benhamou MA Non-drug treatment (excluding surgery) in rheumatoid arthritis: clinical practice guidelines Joint Bone Spine 2009 76 691 698 10.1016/j.jbspin.2009.01.017 19945896

[B9] Gossec L Pavy S Pham T Constantin A Poiraudeau S Combe B Flipo RM Goupille P Le Loët X Mariette X Puéchal X Wendling D Schaeverbeke T Sibilia J Tebib J Cantagrel A Dougados M Nonpharmacological treatments in early rheumatoid arthritis: clinical practice guidelines based on published evidence and expert opinion Joint Bone Spine 2006 73 396 402 10.1016/j.jbspin.2006.01.008 16626995

[B10] Christie A Jamtvedt G Dahm KT Moe RH Haavardsholm EA Hagen KB Effectiveness of nonpharmacological and nonsurgical interventions for patients with rheumatoid arthritis: an overview of systematic reviews Phys Ther 2007 87 1697 1715 10.2522/ptj.20070039 17906290

[B11] Hammond A Rehabilitation in rheumatoid arthritis: a critical review Musculoskeletal Care 2004 2 135 151 10.1002/msc.66 17041978

[B12] Kavuncu V Evcik D Physiotherapy in rheumatoid arthritis MedGenMed 2004 6 3 15266230 PMC1395797

[B13] Zhang W Doherty M Leeb BF Alekseeva L Arden NK Bijlsma JW Dinçer F Dziedzic K Häuselmann HJ Herrero-Beaumont G Kaklamanis P Lohmander S Maheu E Martín-Mola E Pavelka K Punzi L Reiter S Sautner J Smolen J Verbruggen G Zimmermann-Górska I EULAR evidence based recommendations for the management of hand osteoarthritis: report of a Task Force of the EULAR Standing Committee for International Clinical Studies Including Therapeutics (ESCISIT) Ann Rheum Dis 2007 66 377 388 10.1136/ard.2006.062091 17046965 PMC1856004

[B14] Valdes K Marik T A systematic review of conservative interventions for osteoarthritis of the hand J Hand Ther 2010 23 50 334 10.1016/j.jht.2010.05.001 20615662

[B15] Ye L Kalichman L Spittle A Dobson F Bennell K Effects of rehabilitative interventions on pain, function and physical impairments in people with hand osteoarthritis: a systematic review Arthritis Res Ther 2011 13 R28 10.1186/ar3254 21332991 PMC3241372

[B16] Wessel J The effectiveness of hand exercises for persons with rheumatoid arthritis: a systematic review J Hand Ther 2004 17 174 180 10.1197/j.jht.2004.02.006 15162104

[B17] Chadwick A A review of the history of hand exercises in rheumatoid arthritis Musculoskeletal Care 2004 2 29 39 10.1002/msc.54 17041966

[B18] Guzelkucuk U Duman I Taskaynatan MA Dincer K Comparison of therapeutic activities with therapeutic exercises in the rehabilitation of young adult patients with hand injuries J Hand Surg Am 2007 32 1429 1435 10.1016/j.jhsa.2007.08.008 17996780

[B19] Gillen G Stroke Rehabilitation – Function Based Approach 2011 3

[B20] Szturm T Peters JF Otto C Kapadia N Desai A Task-specific rehabilitation of finger–hand function using interactive computer gaming Arch Phys Med Rehabil 2008 89 2213 2217 10.1016/j.apmr.2008.04.021 18996252

[B21] Wilkinson N Ang RP Goh DH Online video game therapy for mental health concerns: a review Int J Soc Psychiatry 2008 54 370 382 10.1177/0020764008091659 18720897

[B22] Wong FS Campbell DR Becker BE Head injury and video games West J Med 1983 138 107 6404061 PMC1010657

[B23] Lange B Flynn SM Rizzo AA Game-based telerehabilitation Eur J Phys Rehabil Med 2009 45 143 151 19282807

[B24] Betker AL Szturm T Moussavi ZK Nett C Video game-based exercises for balance rehabilitation: a single-subject design Arch Phys Med Rehabil 2006 87 1141 1149 10.1016/j.apmr.2006.04.010 16876562

[B25] Szturm T Betker A Goodman V Desai A Moussavi Z Effects of an interactive computer game exercise regimen on balance impairment in frail community dwelling older adults: a randomized controlled trial Phys Ther 2011 91 1 14 10.2522/ptj.20090205 21799138

[B26] Herndon CD Decambre M McKenna PH Interactive computer games for treatment of pelvic floor dysfunction J Urol 2001 166 1893 1898 10.1016/S0022-5347(05)65714-X 11586256

[B27] Higgins S Motor skill acquisition Phys Ther 1991 71 123 139 1989008 10.1093/ptj/71.2.123

[B28] Roddy E Zhang W Doherty M Arden NK Barlow J Birrell F Carr A Chakravarty K Dickson J Hay E Hosie G Hurley M Jordan KM McCarthy C McMurdo M Mockett S O’Reilly S Peat G Pendleton A Richards S Evidence-based recommendations for the role of exercise in the management of osteoarthritis of the hip or knee – the MOVE consensus Rheumatology (Oxford) 2005 44 67 73 10.1093/rheumatology/keh399 15353613

[B29] Bennell LK Hinman SR A review of the clinical evidence for exercise in osteoarthritis of the hip and knee J Sci Med Sport 2011 14 4 9 10.1016/j.jsams.2010.08.002 20851051

[B30] Care for people with arthritis Evidence and Best Practices 2005

[B31] Lancaster GA Dodd S Williamson PR Design and analysis of pilot studies: recommendations for good practice J Eval Clin Pract 2004 10 307 312 10.1111/j..2002.384.doc.x 15189396

[B32] Stamm TA Machold KP Smolen JS Fischer S Redlich K Graninger W Ebner W Erlacher L Joint protection and home hand exercises improve hand function in patients with hand osteoarthritis: a randomized controlled trial Arthritis Rheum 2002 47 44 49 10.1002/art1.10246 11932877

[B33] Brorsson S Hilliges M Sollerman C Nilsdotter A A six-week hand exercise programme improves strength and hand function in patients with rheumatoid arthritis J Rehabil Med 2009 41 338 342 10.2340/16501977-0334 19363566

[B34] Buljina AI Taljanovic MS Avdic DM Hunter TB Physical and exercise therapy for treatment of the rheumatoid hand Arthritis Rheum 2001 45 392 397 10.1002/1529-0131(200108)45:4<392::AID-ART353>3.0.CO;2-2 11501728

[B35] Dellhag B Wollersjo I Bjelle A Effect of active hand exercise and wax bath treatment in rheumatoid arthritis patients Arthritis Care Res 1992 5 87 92 10.1002/art.1790050207 1390969

[B36] Flatt AE Restoration of rheumatoid finger-joint function J Bone Joint Surg Am 1963 45 1101 1103 14046473

[B37] Hoenig H Groff G Pratt K Goldberg E Franck W A randomized controlled trial of home exercise on the rheumatoid hand J Rheumatol 1993 20 785 789 8336303

[B38] Backman C Mackie H Reliability and validity of the Arthritis Hand Function test in adults with osteoarthritis Occup Ther J Res 1997 17 55 67

[B39] Backman C Mackie H Arthritis hand function test: inter-rater reliability among self-trained raters Arthritis Care Res 1995 8 10 15 10.1002/art.1790080105 7794974

[B40] Raven EE Haverkamp D Sierevelt IN van Montfoort DO Poll RG Blankevoort L Tak PP Construct validity and reliability of the disability of arm, shoulder and hand questionnaire for upper extremity complaints in rheumatoid arthritis J Rheumatol 2008 35 2334 2338 10.3899/jrheum.080067 19004045

[B41] Beaton DE Katz JN Fossel AH Wright JG Tarasuk V Bombardier C Measuring the whole or the parts? Validity, reliability & responsiveness of the disabilities of the arm, shoulder, and hand outcome measure in different regions of the upper extremity J Hand Ther 2001 14 128 146 10.1016/S0894-1130(01)80043-0 11382253

[B42] Andersen Hammond ER Shay BL Szturm T Objective evaluation of fine motor manipulation-a new clinical tool J Hand Ther 2009 22 28 35 quiz 36 10.1197/j.jht.2008.06.006 18950989

[B43] Lockery D Peters JF Ramanna S Shay BL Szturm T Store-and-feedforward adaptive gaming system for hand-finger motion tracking in telerehabilitation IEEE Trans Inf Technol Biomed 2011 15 467 473 21536526 10.1109/TITB.2011.2125976

